# The sexuality experience of stoma patients: a meta-ethnography of qualitative research

**DOI:** 10.1186/s12913-023-09532-2

**Published:** 2023-05-16

**Authors:** Siting Lin, Guo Yin, Linghui Chen

**Affiliations:** 1 School of Nursing and Health, Nanfang College Guangzhou, Guangzhou, China; 2 Florence Nightingale Faculty of Nursing, Midwifery & Palliative Care, King’s College, London, UK

**Keywords:** Surgical stoma, Sexual behavior, Qualitative research, Meta-ethnography

## Abstract

**Background:**

As the quality of life of stoma patients has become a research hotspot, sexual health as an integral part of patients’ lives has received more and more attention. However, there is a lack of comprehensive reviews on the sexual experiences of patients with stomas. This study aims to synthesize the qualitative literature on the subjective experience of stoma patients’ sexual life, to identify their sexual needs, and to provide evidence for the content and methods of sexual health interventions for healthcare professionals.

**Methods:**

PubMed, Embase, Web of Science, CINAHL, and Scopus were searched for qualitative studies on the sexual experience of stoma patients (from the inception to January 2023). Titles, abstracts, and full texts were reviewed by two researchers. We used the Critical Appraisal Program (CASP) checklist to assess the quality of included articles.

**Results:**

A total of 1388 articles were retrieved, and eight studies were included. Data was extracted, including three main themes: 1) sexual problems due to changes in physical function and psychological disorders; 2) the relationship with spouse changes; 3) the cognition of sexual life and the need for sexual knowledge.

**Conclusion:**

Healthcare professionals should pay attention to the sexual life status and sexual health needs of stoma patients and their partners, and give professional guidance and support in treatment and nursing to improve the quality of sexual life of stoma patients.

**Supplementary Information:**

The online version contains supplementary material available at 10.1186/s12913-023-09532-2.

## Background

Stomas are frequently utilized in the surgical management of colorectal cancer, inflammatory bowel disease, bladder cancer, and colorectal illness. A stoma is a surgical procedure in which the end of a patient's bowel or ureter is put on the surface of the body to create an opening [1]. Although a stoma is necessary to treat the disease and prolong the patient's survival, stoma surgery is a radical treatment where the anus or urethra are moved to the abdominal wall, and the patient's excretion channel and body structure are altered [2]. Due to the patient’s involuntary excretion of urine and faeces through the abdominal stoma, the stoma patient must cope with several physiological issues, such as nasty smell and stool leakage [3]. In addition, stoma patients must also carry a bag attached to the stoma to collect excreta [4]. This excretion method violates the original physiological function of the patient and affects the reorganization of the body image of the stoma patient. This also inevitably leads to stoma patients experiencing psychological problems such as depression, anxiety, low self-esteem, denial, loneliness, hopelessness, and stigma [5].

Patients’ self-esteem and physical deficits can be a barrier to an intimate relationship, which can severely impact patients’ sexual behavior and function [6]. The findings of multiple studies have also confirmed that most stoma patients have sexual dysfunction after surgery [7, 8]. Sexual dysfunction (SD) is a general term that refers to various persistent or recurrent symptoms with the sexual response that causes patients distress, manifesting as changes in sexual desire, arousal, orgasm, and sexual pain [9]. In women, sexual dysfunction can also manifest as sexual pain associated with vaginal stenosis and dryness [10, 11]. Sexual dysfunction in men can also occur as difficulty in achieving and maintaining an erection or premature or delayed ejaculation [12, 13]. Sexual dysfunction is common in stoma patients but often has been underestimated in post-stoma care. A descriptive study of patients with enterostomy in China has indicated that 63.1% of participants suffered from sexual dysfunction [14]. In colorectal patients, the stoma is one of the major risk factors for the sexual disorder, which is significantly associated with loss of sexual desire, dyspareunia, and reduced vaginal dimension for women [15]. A retrospective study by Costa et al. [16] has reported that 65% of men with rectal cancer who experienced a surgical procedure had erectile dysfunction and 27% on the difficulty in ejaculation. The existence of a stoma is the independent risk factor for the erectile dysfunction [17].

Sexual dysfunction directly impacts life satisfaction [18]. It can also cause emotional relationships between patients and their spouses, leading to family tensions and reducing patients’ quality of life with stomas. Sexual health is an integral part of the patient’s life cycle, and a necessary and appropriate sexual life benefits the patient’s health. For stoma patients, however, sex is considered a private matter, and as such, it is disregarded and not brought up by patients, families, or healthcare professionals [19, 20]. In order to improve the quality of life of patients with a stoma, sexual issues must be identified and addressed.

As the quality of life of stoma patients has become a research hotspot [21], sexual health as an integral part of patients’ lives has received more and more attention. However, there is a lack of comprehensive reviews on the sexual experiences of patients with stomas. The sexual experience of stoma patients is a complex problem, and sexual experience is affected by factors such as disease, treatment, society, and psychology [22]. In order to clarify the sexual problems faced by patients with stomas and to provide a more comprehensive picture of their psychosexual experiences and demands, qualitative synthesis on the sexual experience of patients with stomas is necessary.

Qualitative studies are uniquely positioned to reveal perceptions of the sexual experiences of patients with stoma surgery. Meta-ethnography is a method for synthesizing and interpreting qualitative results from various research since they have relevance to a specific phenomenon [23]. In contrast to the meta-analysis and integrative reviews, meta-ethnography is an interpretive work that is consistent with an interpretivist framework [23], which is of great significance when researchers focus on conceptual or theoretical explanations of a specific phenomenon [24]. However, to our knowledge, there is a lack of meta-ethnography regarding the sexual experiences of patients with stomas. Therefore, this meta-ethnography aims to synthesize the qualitative literature on the subjective experience of stoma patients’ sexual life to gain a deeper understanding of their sexual needs, and to provide a theoretical reference for further development of the sexual health interventions targeting the stoma patients.

## Methods

We conducted a meta-ethnography to synthesize qualitative studies on the sexual experiences of stoma patients. This study followed the eMERGe Meta-ethnography reporting guidance [25] and Preferred Reported Items for Systematic Reviews and Meta-Analyses (PRISMA) [26]. The meta-ethnography has been registered with PROSPERO (ID: CRD42023417096).

### Search strategy

PubMed, Embase, Web of Science, CINAHL, and Scopus were searched from inception to January 2023 using Boolean operators to retrieve the search terms, without time restrictions. The search strategy was demonstrated in the Supplementary File 1. To identify other relevant studies, the electronic searches were supplemented by a manual retrieval of the references of included articles and reviews. SL and GY performed the literature search and study screening.

### Study selection

Studies were included if they met the inclusion criteria: Qualitative research design or mixed-methods studies (i.e., qualitative and quantitative) with qualitative data that could clearly be distinguished; focusing on the sexuality experience of the patients with a stoma. Studies utilizing mixed samples (i.e., stoma patients, spouse/partners, and healthcare professionals) were excluded unless the qualitative data from patients were reported separately [27, 28].

### Reading, data extraction, and quality assessment

**Fig.1:**
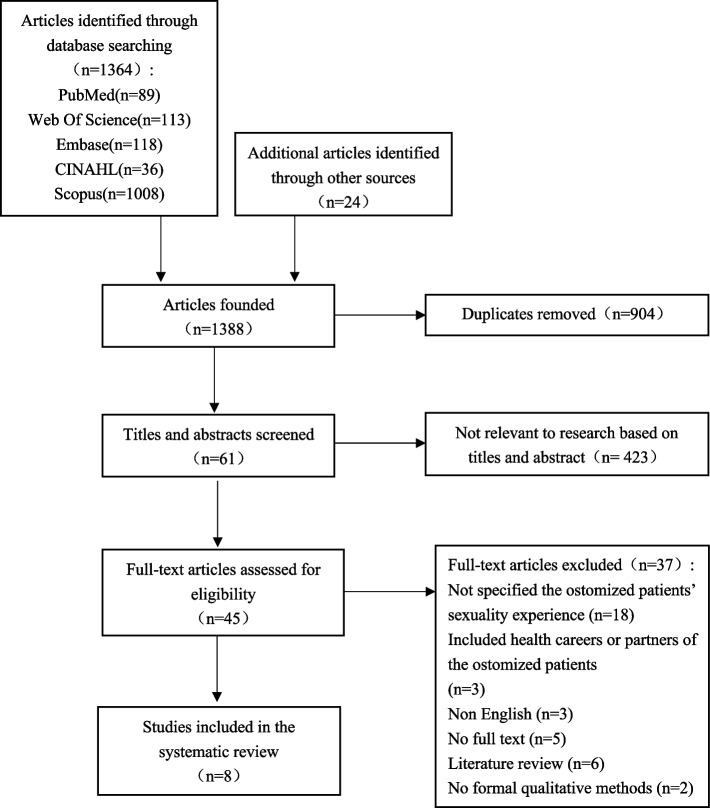
Flowchart of search results and study selection

The research team (SL, GY, and LC) read and re-read the included articles intensely and critically to become acquainted with the detailed content of the papers and kept a reflective record for auditing and reflexivity. The onset of identification of key concepts and key metaphors accompanied the data extraction phase. SL and GY independently conducted the data extraction. Disagreements or uncertainties were resolved through consultation with a third reviewer (LC). The retrieved studies were imported into EndNote software for duplication checking, and the duplicates were excluded. Titles and abstracts were screened. Full texts were reviewed to confirm that they met the inclusion criteria. Figure 1 illustrated the flowchart of studies screening. Data extraction included the following: author, publication year, country, interview scenarios, the number of participants, age range, type of stoma, study design, study aims, main results.

The Critical Appraisal Skills Programme (CASP) checklist was performed for quality assessment of included qualitative studies. Researchers (SL and LC) were independently required to systematically evaluate the quality of a study with ten items by rating “Yes,” “No” or “Can’t tell” for each item. If two-thirds of the ten items were “Yes,” the quality level was rated as “high”; if we got “Yes” for four to six items, it was graded as “Medium” quality; if we got “No” for more than two-thirds of items, the study was considered as “low” quality. Disagreements or uncertainties were resolved through consultation with a third reviewer (GY).

### Determining how the studies are related

The implementation of literature quality assessment contributes to the familiarity with studies. Accordingly, the CASP checklist was also used to determine that included studies are related to the research topic [24]. Although the qualitative methodologies used in the included research were slightly varied, they were comparable regarding data collecting and the recruiting environment. Additionally, they were sufficiently comparable in that they concentrated on the subjective experiences of sexuality for patients with stoma surgery. Further, while the participants in most studies demonstrated different demographic characteristics (such as gender, age, etc.), given the purpose of the meta-ethnography, these characteristics were not deemed relevant. As a result, a reciprocal translation synthesis was made possible by the research that was included [29].

### Translation and qualitative synthesis

The included studies were synthesized using Noblit and Hare’s qualitative research synthesis approach [23]. All researchers repeatedly read all included articles and comparatively analyzed the raw data to identify key metaphors or concepts. Key metaphors were juxtaposed into a grid. Comparing and contrasting the key metaphors of included studies in the grid facilitated the reciprocal translations between the included articles and the synthesis of translations to illuminate more refined concepts.

The third-order construct was used to perform the data synthesis [30]. Key themes reflecting participants’ raw accounts were taken from the “result” segments of included articles to form first-order constructs. The authors’ interpretations of participants’ accounts were used as second-order constructs. Finally, through discussion and interpretation of the first- and second-order constructs, the third-order constructs were identified by reviewers (SL, GY, and LC). First-order themes were independently categorized by the researchers based on primary data from included articles, and then were tabulated into the grid of first-order constructs and used to develop higher-order concepts [31]. These processes were also repeated in the second-order constructs. The second-order interpretations were synthesized into the third-order constructs. All findings were discussed and confirmed among the research group.

## Results

A total of 1388 results were returned from database searches. After the elimination of 904 duplications, 484 studies were identified for the title and abstract screening. Full texts of 61 studies were identified for further screening after the exclusion of 423 irrelevant studies. Finally, eight studies [4, 27, 28, 32–36] met the inclusion criteria and were included in this meta-synthesis. The flowchart was demonstrated in Fig. 1.

### Quality assessment of the Studies

The results of included studies’ quality assessment were provided in Table 1. The eight included studies were of medium to high quality. Six articles indicated high-quality ratings, accounting for 75% of the total number of research [4, 27, 28, 33–35]. All studies provided a clear statement of the purposes and mentioned ethical issues. No studies considered the relationship between the participants and the researcher. All eight studies were included in this meta-synthesis.

**Table 1:** A summary of the CASP critical appraisal results for the included studies in this systematic review

Study	Q1	Q2	Q3	Q4	Q5	Q6	Q7	Q8	Q9	Q10	Score	Classification of Quality
Manderson [32]	Yes	Yes	?	?	?	No	Yes	No	Yes	Yes	13/20	Moderate
Ramirez et al. [4]	Yes	Yes	Yes	Yes	Yes	No	Yes	Yes	Yes	Yes	17/20	High
Paula et al. [33]	Yes	Yes	Yes	No	No	No	Yes	Yes	Yes	Yes	14/20	High
Cardoso et al. [34]	Yes	Yes	Yes	?	Yes	No	Yes	Yes	Yes	Yes	17/20	High
Vural et al. [35]	Yes	Yes	Yes	?	Yes	No	Yes	Yes	Yes	Yes	16/20	High
Kimura et al. [36]	Yes	Yes	Yes	?	No	No	Yes	?	Yes	Yes	13/20	Moderate
Sarabi et al. [28]	Yes	Yes	Yes	?	?	No	Yes	Yes	Yes	Yes	16/20	High
Kandemir and Oskay [27]	Yes	Yes	Yes	?	Yes	No	Yes	?	Yes	Yes	15/20	High

Critical Appraisal Skills Programme (CASP) questions scoring: Yes = 2, ?(Can’t tell) = 1, No = 0

Q1. Was there a clear statement of the aims of the research?

Q2. Is a qualitative methodology appropriate?

Q3. Was the research design appropriate to address the aims of the research?

Q4. Was the recruitment strategy appropriate to the aims of the research?

Q5. Was the data collected in a way that addressed the research issue?

Q6. Has the relationship between researcher and participants been adequately considered?

Q7. Have ethical issues been taken into consideration?

Q8. Was the data analysis sufficiently rigorous?

Q9. Is there a clear statement of findings?

Q10. How valuable is the research?

### Characteristics of the included studies

The total sample of all studies was 194 participants, with sample sizes ranging from 10 to 56 participants. Approximately 58% of the participants were male. All studies were published from 2005 to 2017. The studies originated from Brazil (*n* = 3), Turkey (*n *= 2), the USA (*n* = 1), Australia (*n* = 1), and Iran (*n* = 1). Only six papers reported specifically type of stoma, and we could not determine the proportion of various stomas from the available data. Descriptive qualitative research (*n* = 4), phenomenology (*n* = 2), grounded theory (*n* = 1), and mixed-method designs (*n* = 1) were employed in the included studies. Table 2 presents the characteristics of the included studies.

**Table 2:** Characteristics of the included studies

**Study (Publication year/Country)**	**Setting**	**Number of participants (Males** **/Females)**	**Age range (years)**	**Type of stoma (n)**	**Study design**	**Aim of study**	**Main results**
Manderson [32] (2005/Australia)	Participants’ homes	*N* = 32 (11 M/21F)	24–82	Not reported	Unstructured interviews; descriptive qualitative research; thematic analysis	Explore men and women’s experiences of adapting to a stoma	Three themes: Adapting to changes; disguise and discomfort; sex and sexiness
Ramirez et al. [4] (2009/USA)	Participants’ home, local medical facility, or most convenient location	*N* = 30 (0 M/30F)	44–93	Not reported	Semi-structured, open-ended interviews; grounded theory; grounded theory approach	Explore the experiences related to sexuality among female colorectal cancer survivors with permanent intestinal stomas	Four themes: No long-term sexual difficulties: Long-term sexual difficulties; Age-related changes in sexuality; No partnered sexual experience post-surgery
Paula et al. [33] (2012/Brazil)	Not reported	*N* = 15 (7 M/8F)	≥ 30	Colostomy (12), ileostomy (2), loop transvers ostomy (1)	Semi-structured interviews; descriptive qualitative research; content analysis	Identify Social Representations of people with intestinal stoma regarding how they experience sexuality before and after the stoma production	Resulting in the thematic unit “Giving new meaning to sexuality” and subthemes: changes in how to experience sexuality after the stoma; new concerns related to the sexual ac; strategies to adapt moments of intimacy; the technique of irrigation and use of intestinal occluder as factors that facilitate the sexual act; the concern about not damaging the stoma; the sexual disorders or dysfunctions resulting from the surgery; the possibility of fully experiencing sexuality despite the stoma and the distance kept form sex as an option
Cardoso et al. [34] (2015/Brazil)	an available and convenient unit in the private room for participants	*N* = 10 (6 M/4F)	≥ 60	Colostomy (9), ileostomy (1)	Semi-structured interview; descriptive qualitative research; inductive content analysis	describe the experience of sexuality and other everyday life aspects for people with intestinal stoma	Three themes: Physical, emotional, and socio-cultural changes; Changes in the exercise of sexuality of people with intestinal ostomy; importance of the interdisciplinary support of the new sexuality
Vural et al. [35] (2016/Turkey)	A quiet, well-lightened, and aerated room without a telephone or other distracting technologies	*N* = 14 (7 M/7F)	28–56	Colostomy (7), ileostomy (6), urostomy (1)	Unstructured interviews; phenomenological qualitative design; content analysis	Describe the lived experiences of persons with stomas related to sexual function and perceptions and their expectations of the stoma nurses who care for them	Five themes: changes in sexual life; changes in body image; fear and anxiety experienced during sexual intercourse; psychological impact of sexual problems; and expectations concerning sexual counseling from ostomy nurses
Kimura et al. [36] (2017/Brazil)	Not reported	N = 56 (56 M/0F)	20–70	Not reported	Mixed-methods studies, qualitative and quantitative; individual interviews; Bardin’s content analysis	Analyze the perception of ostomized men due to intestinal cancer regarding sexual relations as an important dimension of quality of life	Five themes: ostomy, self-care, acceptance, self-concept, and companionship
Sarabi et al. [28] (2017/Iran)	At the park, participants’ homes, airport or in the Iranian Ostomy Association	*N* = 27 (15 M/12F)	24–74	Colostomy (11), ileostomy (10), urostomy (6)	Unstructured interviews; descriptive qualitative research; inductive content analysis	Explore the sexual performance experiences of patients with a stoma and their spouse	Five themes: experiencing sexual problems; patient’s reaction to sexual problems; confronting with consequence of sexual problems; reproductive difficulties and facing with the effects of the menopause
Kandemir and Oskay [27] (2017/Turkey)	A quiet room	*N* = 10 (10 M/0F)	Not reported	Urostomy (10)	Open-ended interviews; phenomenological qualitative design; content analysis	Identify experiences, views, and problems of bladder cancer patients with urostomy regarding the effects of urostomy on their and their spouse or partner’s sexual life	Three themes: changes in body image; problems experienced during sexual activity; and receiving help and support in relation to the problems experienced

### Data analysis and synthesis

Synthesis of qualitative research generated the first-, second-, and third-order constructs that constitute the foundation of meta-ethnography. Twenty-four first-order concepts were identified from the original data of participants, and six second-order concepts were further determined to interpret the first-order constructs, including physiological changes in sexuality, psychological changes in sexuality, the changes in communication with spouses, the changes in intimacy relationship with spouse, patients’ perception of sexuality, and patients’ needs for knowledge about sexual health. Three overarching themes were finally generated through the synthesis of the second-order interpretations. Table 3 presents the synthesis findings of the first-, second-, and third-order constructs. Three overarching themes were as follows:

- sexual problems due to changes in physical function and psychological disorders
- the relationship with the spouse changes
- the cognition of sexual life and the need for sexual knowledge

**Table 3:** Meta-ethnography translation table

Third-order constructs	Second-order constructs	First-order constructs	Source study numbers
Sexual problems due to changes in physical function and psychological disorders	Physiological Changes in sexuality	Sexual dysfunction	1, 3, 4, 5, 7, 8
		Pain during intercourse	1, 2, 3, 4, 7, 8
		The decrease in sexual desire	1, 2, 5, 7, 8
		The decrease in sexual attraction	1, 2, 5, 6
		Impediment of sexual activity	1, 2, 3, 4, 5, 6
	Psychological changes in sexuality	Feeling of embarrassment	1, 3, 4, 5, 7
		Feeling of guilty	7
		Becoming irritable	7
		Low self-esteem	4, 5, 6
		Body image disorders	1, 2, 3, 4, 5, 6, 7, 8
		Resistance to physical exposure with partners	1, 2, 3, 4, 6, 8
The relationship with spouses Changes	The changes in communication with spouses	Lack of communication with spouse	3, 6, 7
		Difficulty getting along with spouse	4, 5, 7
		The importance of companionship and support from partner	2, 6, 7
	The changes in intimacy relationship with spouse	Concerns about having sex	1, 2, 3, 4, 5, 6, 8
		changes in sexual attitudes of spouse	1, 8
		The end of a long-term sexual relationship	1, 2, 3, 4, 5, 7, 8
		Finding alternative ways to improve sexual behavior	2, 3, 7, 8
		No change in intimacy relationship	1, 7
The cognition of sexual life and the need for sexual knowledge	Patients’ perception of sexuality	The importance of sexuality	1, 2, 3, 7, 8
		The importance of companionship and non-sexual intimacy of partner	1, 2, 7
	Patients’ needs for knowledge about sexual health	The needs to be informed preoperatively about potential sexual problems of post-stoma	2, 8
		Lack of knowledge about sexuality	2, 3, 4, 5, 7, 8
		The needs for receiving professional guidance on sexual health	1, 2, 3, 4, 7

1 (Manderson [32]); 2 (Ramirez et al. [4]); 3 (Paula et al. [33]); 4 (Cardoso et al. [34]); 5 (Vural et al. [35]); 6(Kimura et al. [36]); 7 (Sarabi et al. [28]); 8 (Kandemir and Oskay [27])

#### Sexual problems due to changes in physical function and psychological disorders

##### Physiological changes in sexuality

All eight papers reported physiological changes in the sexuality of patients with a stoma, mainly including sexual dysfunction, painful intercourse, decrease in sexual desire, hindered sexual activity, and loss of sexual attractiveness [4, 27, 28, 32–36].

**Sexual dysfunction:** The autonomic nervous system from the pelvis to the sexual organs of the stoma patients may be damaged due to the abdominoperineal surgery, which can lead to sexual dysfunction such as loss of ejaculation or retrograde ejaculation in men and dryness of the vagina and discomfort during sexual activity in women. When stoma patients were asked to describe changes in their sexual function after stoma surgery, responses stated, *“Now I have erection, but no ejaculation.”* [33] *“There is a vaginal stenosis and in the last two intercourses. I had so much pain and distress…I have so much vaginal dryness…” *[35]*.*

**Pain during intercourse:** After stoma surgery, some patients reported pain during sexual intercourse with their partner, which affected the enjoyment of sex for both patients and their partners.* “When he knows it hurts, it’s in the back of his mind constantly. I know that makes it more difficult for him to have an orgasm…” *[4]*.* *“I have so much fear. I felt pain because my vagina was cut and sewn.” *[28].

**Decrease in sexual desire:** Surgery and treatments like chemotherapy and radiotherapy negatively affect the entire body of stoma patients, which can cause pain and discomfort during sexual activity and reduce sexual desire. *“My sex drive was too low. Chemotherapy affects the whole body and reduces a man’s sexual desire unlike a healthy man” *[28] The change in bowel movements after stoma surgery makes it difficult for stoma patients to control disposal during sexual intercourse, resulting in an unpleasant or embarrassing sexual experience and a loss of sexual desire. *“It was just the embarrassment. As happened a few times that it had unpleasant sound or began to work because I could not control it. I became frigid” *[28].

**Decrease in sexual attraction:** Changes in body image from stoma surgery and the inability to dispose of feces and gas reduced the patient’s perception of their sexual attraction and no longer made them desirable sexual partners. *“I don’t find myself attractive. I don’t like and concentrate and as the stoma stays there, I don’t want to have sex…” *[35].

**Impediment of sexual activity:** The patient’s use of a stoma bag to collect faeces, which was a barrier that impaired the patient’s sexual experience during sexual intercourse, as well as changes to their body image as a result of the stoma surgery. *“If we do it in the missionary position, the bag plastic material disturbs a lot.” *[33].

**Psychological changes in sexuality**

Patients with stomas experienced psychological changes as well as unpleasant psychosexual emotions as a result of the altered body shape brought on by the stoma surgery and the lack of control over bowel excretion and odors.

**Feeling of embarrassment:** Some patients reported embarrassment when engaging in sexuality with their spouse because of the sounds associated with the stoma and failing to control disposal. *“After surgery I became cold in terms of sexual relations… I did not have sexual problem, it was just the embarrassment”* [28].

**Feeling of guilty:** Due to the sexual dysfunction following stoma surgery, some patients felt guilty toward their partners and expressed emotional distress such as anxiety, depression, and sadness about their sexual abilities and responsibilities for not being able to satisfy the sexual needs of their partners. *“I feel guilty that I cannot meet her demands. I feel that I am a burden for her” * [28].* “My responsibilities both for my wife and myself compel deeply.” *[35].

**Becoming irritable:** Some of the stoma patients’ partners reported that they became irritable after the surgery, *“When my husband was impotent he became bad-tempered,” “My wife has become so irritable after the surgery.”* [28].

**Low self-esteem:** Stomas affected patients’ body image, undermining the idealized vision of the perfect woman and society’s expectations of women, leading to low self-esteem in female patients. *“I feel diminished about everything… For other women, I feel greatly diminished”* [34].

**Body image disorders:** Patients with stomas found it challenging to adapt to the altered body image after stoma surgery, such as the presence of stoma bag and fecal dirt, discomfort with the appearance of stoma, and the unpleasant odor, and therefore experienced body image disorders. Most stoma patients reported dissatisfaction with their self-image after a stoma. *“this thing hanging there, that I would just like to rip it off and throw it away, you know, if I could.” *[32]. Some patients even reported that they had difficulty reconciling their disgust with the stoma and the distress caused by it. *“I smell bad to me, so it’s bad to me…in ways I haven’t quite sorted out” *[4].

**Resistance to physical exposure with partners:** The physical changes that came with a stoma also made patients resistant to physical exposure with their partners. The stoma represented a loss of physical integrity. Exposure to “the altered body” would make the patients perceive themselves as becoming unattractive sexually, leading to reluctance to be seen naked by their partners. *“After the surgery, I never let my wife see me naked again. I feel I am not good as a man anymore”* [36]. Stoma patients preferred to seek ways of concealment or to cover up their stoma pouch during sexual activity [32]. Several female patients reported covering the stoma with a towel or nightgown before sexual activity to keep the stoma bag stable and avoid possible leakage during sexual intercourse. Moreover, they did not want to see the stoma bag during sexuality, considering that the presence of feces is contrary to sexual desire in our culture of fecal aversion. *“Sometimes he'll want to have sex and I have a little bowel in my bag and I'll excuse myself and get another bag, because I don't want him to feel that when he's lying on top of me. That would make me feel gross” *[4].

#### The relationship with the spouse changes

**The changes in communication with partner**

**Lack of communication with spouse: **Communication with a partner is an important part of healthy sexual life which is directly related to the couple’s emotions and contributes positively to the maintenance of a successful partnership [33]. However, in reality, many stoma patients and their spouses avoided discussing sexuality, and there was a dearth of contact and discourse between the couple. They grew farther distant from one another on account of the stoma. *“Now we sleep separate bedrooms. At home we do not have much to talk about”* [28].

**Difficulty getting along with spouse:** Sexual problems and emotional distress caused by the stoma changed the couple’s relationship, resulting in turbulent married life. Some patients reported that there were difficulties in getting along with their partners. *“I had a partner. She supported me but then, I did not want her anymore… I got sick of her. I did not want to see her face” *[35].

**The importance of companionship and support from partners:** Stoma patients expressed the importance of companionship and support from their partners. *“Without my wife, I could never live with this pouch,” “My wife made me feel like a better man”* [36]. Sexual-related companionship is considered an important factor in the sexual relationship between couples. Respect, companionship, and mutual love are necessary for a sexual relationship between couples. *“Because you needs to be with someone who respects you, she is affectionate, who likes you as you are…” *[34]*.*

**The changes in intimacy relationship with spouse**

Stoma surgery placed a heavy burden on the sexual relationship of most patients and their partners, resulting in family conflicts such as sexual incompatibility and affecting marriage life.

**Concerns about having sex:** Some patients reported that after the stoma, they and their partners had concerns about having sex, how it might affect the stoma or any accidents that might occur during sexual activity. *“I was afraid that the stoma bag might tear when we cuddle and when we were intercourse.” *[35]* “At the beginning, I always avoided sexual intercourse because I was worried about hurting my husband.” *[28]

**Changes in sexual attitudes of spouse:**Some patients’ partners experienced changes in sexual attitudes due to their altered bodies, such as loss of sexual desire and even avoiding sexual activity [27]. The sounds, sights, smells, and contact with the stoma and its contents could impair the individual’s sexual desire. *“My husband has very low libido anyway unfortunately”* [32]. *“She finds the bag repulsive and does not like the sight of my hernia or my body”* [32]. Some spouses who took on the responsibility of providing care for the stoma patients struggled to make the change from lovers towards carers. The expense of providing for a partner was sexual withdrawal [32].

**The end of a long-term sexual relationship:** Many couples experienced sexual problems during intercourse, such as sexual dysfunction, pain during intercourse, fear of injury to their spouse, and interference with the stoma. They affected the couple’s enjoyment of sex and caused their sexual relationship to become frigid, resulting in separation or divorce and the end of a long-term sexual relationship. *“The one thing that we used to love to do together and it's kind of gone. And it's been gone for a long time” *[4]. *“We have been sleeping in separate beds for three years. I have sexual desire, but I cannot have an erection”* [27].

**Finding alternative ways to improve sexual behavior:** Many patients expressed that they sought to find alternative ways to improve their sexual behavior with their partners. For example, changes in sexual approach (replacing traditional intercourse with other intimate acts), *“I am satisfied with actions such as kissing, stroking, *etc*. as well. However, I do not ejaculate. My wife lets me stroke her because I want so”* [27]; cleaning and securing the stoma bag before sexual intercourse, *“at the moment of sex, I LET THE BAG CLEAN AND WRAP IT WITH MICROPORE, then, it doesn’t disturb at all”* [33]; adopting more comfortable sexual positions, *“The traditional position is uncomfortable for both two people, changing the position… not to bring harm, nuisance and *vice versa*”* [34].

**No change in intimacy relationship:** However, some patients reported no change in their intimacy relationship with their partner after stoma surgery, *“Our relationship hasn’t altered, not our sex life. He doesn’t ‘cringe’ if he touches the bag on my tummy when we make love; it is just as if it wasn’t there”* [32]. Having a deep emotional connection between the couple as a bond sometimes can make the partners choose to accept the sexual problems brought up by the stoma.*“When you love your wife there’ll be no frigidity, no distance. We love each other…We’re satisfied by touching each other. We never quarrel” *[28]*.*

#### The cognition of sexual life and the need for sexual knowledge

**Patient’s perceptions of sexuality**

**The importance of sexuality:** Sexuality is a normal physiological need of the individual. Both stoma patients and their partners had regular sexual needs, *“I was younger, seeking to be horny, to have best horny feeling.”* [33] However, they were unable to have their sexual need met because of the sexual problems associated with their stoma. *“Every woman needs to have sex, to flirt, with her husband…but now we cannot”* [28]. Some patients also expressed that they regretted the changes to their sexual life that their stoma has brought about. *“Sexuality was important for me, if I had had another option, I would never have had this operation…I want my former life back.” *[27].

**The importance of companionship and non-sexual intimacy of partner:** Some patients claimed to have reached an understanding with the thought of no longer engaging in sexual activity. They valued their partners’ companionship and non-sexual intimacy as a crucial component of their present intimate relationships. The importance of sexuality inside the marriage had diminished from its former prominence in one’s earlier years. *“There is more intimacy at the mental level than at the physical level…Just physical closeness is a good thing… just holding hands, sitting together watching TV” *[4].

**Patients’ needs for knowledge about sexual health**

**The needs to be informed preoperatively about potential sexual problems of post-stoma:**Stoma patients need to be informed about sexual problems that may develop after stoma surgery in the preoperative period, as this may affect their treatment options and contribute to managing their postoperative expectations [37]. “*Sexuality was important for me, if I had had another option, I would never have had this operation…I want my former life back.*” [27].

**Lack of knowledge about sexuality:** Stoma patients reported a lack of knowledge about sexuality after surgery. Patients were unaware of the possibility of resuming sexual intercourse after treatment. *"We had no intercourse……A year after chemotherapy, I was scared that my condition might have gotten worse"* [28]. Their partners were concerned that sex after a stoma might adversely affect the patient’s health, so they avoided sexual intercourse. *“I avoided sexual intercourse for a long time in order not to hurt him…My husband’s health was more important than anything else to me” *[27].

**The needs for receiving professional guidance on sexual health:** Stoma patients shared expectations about receiving professional guidance on sexual health from healthcare professionals. *“With guidance (sexuality), I would feel safer, for sure”* [34]. Some patients reported that they would also like their partners to have access to professional guidance. *“Patient but especially the spouse must be informed” *[35]. Sexuality is a personal and intimate subject. Patients rarely consulted with healthcare professionals about sexual guidance for fear of embarrassment or because they were unsure whether it was appropriate to discuss this topic during treatment. *“But we feel shy. We cannot talk to anyone; we even do not know how to ask such thing”* [27]. At the same time, healthcare professionals rarely provided patients with professional guidance on sexuality-related issues. *“Till today no one guided or asked anything about sexuality but you” *[35]. The majority of people acknowledged their need for expert advice from healthcare professionals and thought the information they gave was helpful. *“I find guidance on sexuality important……the more information, the more the person’s head improves….” *[34].

## Discussion

This study synthesized qualitative research on the sexual experiences of stoma patients. The integrated themes of this review contribute to understanding the post-stoma sexual experience from the perspective of the stoma patients. Capilla-Díaz et al. [38] previously performed a systematic review of qualitative research on the experiences of living with a stoma. However, this review did not focus on the experiences related to stoma patients’ sexuality, as sexuality is an essential and non-negligible part of stoma patients’ life. In this study, we found that patients with stoma experienced shifts in sexual physiology and sexual psychology, along with changes in relationships with their partners. They also required scientific guidance on sexual health. It is necessary to prompt healthcare professionals to understand further the stoma patient’s emotional experiences and needs related to sexuality.

### The resolution of sexual problems

According to this review, having a stoma generated psychological and physiological issues with sexuality that affected the patient and their partner’s potential to have healthy sexual relationships [22]. From the perspective of sexual physiology, most of the changes in the sexual behavior of stoma patients are caused by stoma surgery or radiotherapy and chemotherapy for colorectal cancer patients. These treatments may deteriorate the stoma patient’s abdominal-pelvic nerves and cause sexual problems, including decreased libido, painful intercourse, erectile dysfunction or retrograde ejaculation in men, and vaginal dryness in women [27, 28, 39]. A stoma will inevitably deteriorate the patient’s body image from the psychosexual aspect. Moreover, the presence of the stoma pouch, the leakage of contents in the stoma, the smell, and the loss of control of bowel excretion can cause psychological distress to stoma patients. In this way, the patient’s self-esteem and self-confidence decrease [34, 36]. Patients feel they are no longer desirable sexual partners, so they are reluctant to allow their partner to see them naked or even resist intimate contact with their partner [32, 33, 36]. These changes are detrimental to the sexual relationship between couples and lead to a decreased quality of sexual life.

The findings of this analysis show that recognizing changes in stoma patients’ sexual physiology and psychology and developing tailored intervention strategies by healthcare professionals are key to helping them resume satisfying sexual relationships. Healthcare professionals can use cognitive behavioral therapy or sensate focus to improve the problem of low libido in stoma patients; use vacuum constriction devices or penile injections to improve erectile dysfunction in male patients; use local estrogen therapy and pelvic floor physical treatment to improve vaginal dryness and pain during intercourse in female patients [40]. These methods improve the impact on patients with sexual physiological distress caused by stoma surgery and treatments. Moreover, the control and safety of the stoma can also be enhanced by emptying the stoma bag before sexual activity and using opaque stoma bags or stoma shutters during intercourse [22]. Furthermore, using lavender essential oil during sexuality reduces stoma odor and helps patients adapt to the stoma [41]. In these way, the patient’s sexual attraction, self-esteem, and self-confidence significantly improve.

The studies on psychological intervention for stoma patients are limited in number. Healthcare professionals often pay more attention to the physical needs of stoma patients while ignoring their psychological needs. This was also mentioned by Ayaz-Alkaya [5], whose research echoed the ignorance of interventions to improve psychosocial problems in stoma patients. A study of a psycho-educational intervention trial with female rectal and anal cancer patients indicated that this intervention positively improved the sexual function and psychological status of patients [42].. In this study, we suggest that healthcare professionals should be committed to providing more psychological interventions to patients with a stoma to help improve their psychological distress and enhance the quality of sexual life for patients and their partners.

### The establishment of a harmonious couple relationship

This ethnography indicated that stoma patients are often reluctant to initiate conversations about sexuality and lack communication with their partners about sexual issues. This finding was also mentioned by Du et al., [43] who indicated that the self-disclosure of patients with colorectal cancer enterostomy is low, and they seldom express their true inner thoughts to others, resulting in a lack of effective communication with their partners. The existence of a stoma and the physical and psychological distress caused by a stoma may affect the couple's relationship. The lack of communication will exacerbate the intimate relationship between the couple, leading to arguments and turmoil in marital life and making it difficult for the patient and partner to get along [28]. This result was accordant with that of Li’s research [44], who concluded that lack of communication could have a negative impact on the couple’s sexual relationship. According to the study by Chang’s research [45], open discussion between couples can facilitate sexual expression and intimate relationships. Therefore, how to encourage communication between stoma patients and their partners is a crucial topic that requires attention from healthcare professionals.

At this stage, more research focus on intervention strategies for communication with cancer patients, but few studies focus on interventions for stoma patients. Fuoto et al. [46] have used the COMFORT Model to strengthen the communication between nurses, patients, and family members through 4-h communication training and live role-playing, significantly enhancing patients’ communication skills with their families and improving their communication satisfaction. Beach et al. [47] implemented an educational intervention for cancer patients and their family members, making them watch the Cancer Play and guiding them to communicate with each other and express their opinions through storytelling and role-playing. According to this research, watching plays provided an opportunity for patients and their families to communicate on sensitive topics, significantly improving their communication skills. Based on the findings from this review, we suggested that healthcare professionals could conduct various activities (such as communication training, role-playing, and watching entertaining videos) for stoma patients and their partners in order to strengthen their awareness of active communication, broaden their communication channels, and improve their communication skills.

Stoma also adds to the concerns of the patient and partner during intercourse, which significantly affects the sexual experience of the couple. There is a shift in the attitude of some partners toward sex. They do not understand the changes in their spouse’s physical image (such as the appearance of the stoma, the smell, and the leakage of stoma contents), and they often resort to avoiding sex to cope with it. As a result, the dilemma of ending a long-term sexual relationship may occur between patients and their partners. It is widely accepted that sexual relationship is essential to human beings’ physical, mental, and emotional well-being. Sexuality is considered a measure of an individual’s sense of well-being, fulfillment, and security. Moreover, as a measure of a couple’s well-being, harmonious sexual life is conducive to fostering a positive couple relationship [48]. On the contrary, sexual disorders may jeopardize family relationships [49]. Given that, it is crucial for stoma sufferers to discuss their sexual issues and receive the support they need. One of the main strategies in the treatment plan for supporting and improving the sexual health of individuals with ostomies is the attention that healthcare professionals pay to sexuality.

However, the current research focuses more on the intervention of the sexual problems of cancer patients, and little attention has been paid to the intervention strategies for the sexual problems of stoma patients. Reese [50] adopted a couple-centered intervention method to improve the intimate relationship of couples by educating patients and partners about sexual knowledge, which included distributing popular science materials, encouraging couples to engage in intimate activities, and communicating on sensitive topics. The results showed that this intervention method effectively addressed patients’ sexual problems. Therefore, various forms of interventions should be applied to stoma patients and their partners by healthcare professionals, including education on sexual knowledge and psychosexual counseling [51].

The findings of this review also revealed that some patients’ spouses, who have an in-depth emotional connection with their partners, might choose to accept the changes brought about by the stoma and engage in non-sexual behaviors to satisfy their sexual needs. They value emotional communication with each other in marriage rather than sex, which also kept their intimate relationship unaffected by the stoma. Moreover, Tat’s [52] research also concluded that patients and their partners negotiated intimate relationships through non-sexual behaviors, like hugging, touching, or kissing, which increased the sense of support and security perceived by patients and their partners and enhanced their intimacy with each other. Given that, encouraging patients and partners to strengthen non-sexual intimacy is a concernful topic that deserves the attention of healthcare professionals.

### Professional guidance and support from healthcare professionals

The majority of stoma patients agreed on the value of sexual life, but they lacked scientific sexual knowledge related to post-ostomy surgery. While many patients who need information on sexual health are willing to discuss their problems and hope to get expert advice on sexual health from healthcare professionals, few will actually ask for assistance. Additionally, it is uncommon for healthcare professionals to provide guidance to stoma patients and their partners regarding sexual health. This finding was also mentioned in the study by Dames [37], which concluded that many patients highlighted the difficulty of obtaining information about the impact of surgical treatments on their sexual lives, and they were given little advice about sexuality from healthcare professionals. At the same time, issues related to sexuality are not discussed between healthcare professionals and patients unless the patients bring up the relevant topic, so they must seek alternative sources of information, such as through social media, to obtain guidance on sexuality-related issues. The findings of Zhu’s research also showed that stoma-related sexual health education improved the quality of patients’ post-operative sexual lives and that 44% of patients reported that they were in significant need of guidance on sexual life [53]. Therefore, it may be helpful to provide appropriate guidance to improve the quality of sexual life for stoma patients and their partners.

The findings of this study revealed that stoma patients were eager to learn more before having stoma surgery about the potential long-term effects of disease treatment on their post-operative sexual life. Patients also believed that early access to accurate information about potential changes and complications after surgery would help them adjust to post-ostomy life [4, 33]. Healthcare professionals should take the initiative to inquire about the need for guidance on the sexual life of patients before stoma surgery and use various methods (such as the internet, brochures, and community) to broaden the sources of sexual information. Meanwhile, post-operative telephone counseling for stoma patients can be carried out after surgery. In this way, it may be achievable that the patient’s demand for sexual health knowledge can be met, and the patient’s post-operative sexual life can be improved.

It should be noted that stoma-related health professionals also need to be educated on specialized sexuality-related knowledge, including preoperative and postoperative assessment of sexual function, potential sexual problems that post-stoma surgery occur, and specialized sexual counseling after stoma surgery. This is of great significance for satisfying the stoma patients’ needs for sexual guidance and further enhancing their post-stoma quality of sexual life. In Sutsunbuloglu and Vural’s study [20], 79.0% of patients had not been informed about possible sexual problems following stoma surgery, and most patients shared their sexual problems with no one. This indicated that patients with stoma received insufficient information regarding the sexual issues that post-stoma surgery may occur, which may be related to healthcare professionals’ lack of capability to provide specialized sexual guidance [54]. It is clear that healthcare professionals are not well-prepared for dealing with sexual problems in stoma patients, and there is a lack of space for discussion on sexuality in their professional training programs [54]. However, helping stoma patients resume their sexual life within the altered condition demands health professionals’ sexuality-related expertise and skills to propose adequate support and tackle the issue.

Meanwhile, it should be emphasized that it is vital in the practice setting for healthcare professionals, including stoma care nurses, to evaluate the sexual function of stoma patients preoperatively and counsel them about the potential risk of sexual problems that post-stoma surgery occurs, as the preoperative evaluation of sexual functions for patients plays an essential role in establishing their appropriate expectations about postoperative sexual function and quality of sexual life [55]. Also, it should be encouraged for healthcare professionals to perform the postoperative follow-up assessment of sexual function using reliable and valid scales, e.g., the International Index of Erectile Function Questionnaire (IIEF), the Female Sexual Function Index (FSFI), or sex therapy models, e.g., the 5A’s model for sexual health communication with patients [56], so as to early identify the potential risk of sexual issues related to stoma surgery and provide tailored treatment plans to facilitate stoma patients resuming their sexual life.

Furthermore, our results also support the need for postoperative sexual counseling for patients with stomas [35]. There are data indicating that the implementation of individualized sexual counseling contributes to the improvement of sexual function [57]. Hence, it would be necessary for healthcare professionals to promote tailored sexual counseling in the postoperative period for those with a stoma, including treatment options for potential sexual problems (e.g., vaginal dilatators for vaginal stenosis [11], vaginal moisturizers/lubricants for vaginal dryness [58], vacuum constriction devices or penile injections for ED [40]) and options for a more enjoyable sex life (e.g., alternant positions, sex toys, lubricants).

Management and resumption of sexual dysfunction after stoma surgery may be a highly complicated process demanding multidisciplinary cooperation. Given that, the pre-and postoperative evaluation and sexual counseling should be provided by a multidisciplinary team composed of clinical stoma care nurse, sex therapists, urologists, and psychologists, etc. in order to cover all aspects regarding stoma care, psychosexual care, evaluation and resumption of sexual function. Notably, clinical stoma care nurses should play an essential role as primary implementers in providing individualized sexual counseling for patients with a stoma, with the multidisciplinary team providing guidance when necessary.

### Limitations of this study

There are some limitations to this research. First, due to language limitations, only English databases were searched in this study, excluding non-English studies, which may have biased the findings. Second, the included studies were all derived from cross-sectional interviews, with no longitudinal studies. Therefore, further exploration of how the sexual experience of stoma patients changed over time is needed in future studies. Third, there is relatively little literature evidence from Southeast Asia. Considering some differences in sexual culture and ideology between Southeast Asia, Europe, and the United States, it is necessary to increase research on Southeast Asia in the future. Forth, only two studies of the included articles analyzed the responses of stoma patients’ spouses to sexual problems [27, 28]. Further research should include more spouses of stoma patients to enrich the findings.

## Conclusion

A Meta-integrated method was used to systematically evaluate the qualitative studies on the sexual life experience of patients with a stoma. The integrated findings illustrate that stoma patients’ sexual physiology and psyche have changed. The emotional relationship between stoma patients and their spouses might also be impacted by the sexual relationship. Additionally, most patients are aware of the value of sexuality and the need for education about sexual health. However, in most cases, patients rarely seek professional guidance or support from healthcare professionals, and healthcare professionals also rarely provide relevant sexual guidance to patients. Therefore, healthcare professionals must implement personalized education and guidance for sexual problems in stoma patients. In future research, interventions that healthcare professionals can use to provide coping strategies, information, and resources to meet the patients’ sexual needs and improve their quality of sexual life should be developed.

## Supplementary Information

**Additional file 1.**

## Data Availability

The dataset used and analyzed during the current study are available from the corresponding author upon reasonable request.

## References

[1] Shabbir J, Britton DC (2010). Stoma complications: a literature overview. Colorectal Dis.

[2] Kelly O'Flynn S (2016). Protecting peristomal skin: a guide to conditions and treatments. Gastrointest Nurs.

[3] O’Flynn SK (2018). Care of the stoma: complications and treatments. Br J Commun Nurs.

[4] Ramirez M, McMullen C, Grant M (2009). Figuring out sex in a reconfigured body: experiences of female colorectal cancer survivors with ostomies. Women Health.

[5] Ayaz-Alkaya S (2019). Overview of psychosocial problems in individuals with stoma: a review of literature. Int Wound J.

[6] Thorpe G, McArthur M (2017). Social adaptation following intestinal stoma formation in people living at home: a longitudinal phenomenological study. Disabil Rehabil.

[7] Nam SY, Lee H, Kim S, Lee RA (2018). Factors affecting body image and sexual life for the colorectal cancer patients with stoma. Asian Oncol Nurs.

[8] Traa MJ, De Vries J, Roukema JA, Den Oudsten BL (2012). Sexual (dys)function and the quality of sexual life in patients with colorectal cancer: a systematic review. Ann Oncol.

[9] Mimoun S, Wylie K (2009). Female sexual dysfunctions: definitions and classification. Maturitas.

[10] Waetjen LE, Johnson WO, Xing G (2022). Patterns of sexual activity and the development of sexual pain across the menopausal transition. Obstet Gynecol.

[11] Liu M, Juravic M, Mazza G, Krychman ML (2021). Vaginal dilators: issues and answers. Sex Med Rev.

[12] Salonia A, Bettocchi C, Boeri L (2021). European Association of Urology guidelines on sexual and reproductive health—2021 update: male sexual dysfunction. Eur Urol.

[13] Montorsi F, Adaikan G, Becher E (2010). Summary of the recommendations on sexual dysfunctions in men. J Sex Med.

[14] Qin F, Ye X, Wei H (2019). Sexual experience and stigma among Chinese patients with an enterostomy: a cross-sectional, descriptive study. Wound Manag Prevent.

[15] Thyø A, Elfeki H, Laurberg S, Emmertsen K (2019). Female sexual problems after treatment for colorectal cancer–a population-based study. Colorectal Dis.

[16] Costa P, Cardoso JM, Louro H (2018). Impact on sexual function of surgical treatment in rectal cancer. Int Braz J Urol.

[17] Laurberg JR, Laurberg VR, Elfeki H, Jensen JB, Emmertsen KJ (2021). Male erectile function after treatment for colorectal cancer: a population-based cross-sectional study. Colorectal Dis.

[18] Bahayi K, Attaallah W, Yardımcı S, Bulut H, Özten E. Depression, Anxiety, Sexual Dysfunction and Quality of Life in Patients with Ileostomy or Colostomy. Turk J Colorectal Dis. 2018;28(2). 10.4274/tjcd.87369.

[19] Saracco C, Rastelli G, Roveron G, Ferrara F (2019). Sexual function in patients with stoma and its consideration among their caregivers: a cross-sectional study. Sex Disabil.

[20] Sutsunbuloglu E, Vural F (2018). Evaluation of sexual satisfaction and function in patients following stoma surgery: a descriptive study. Sex Disabil.

[21] Näsvall P, Dahlstrand U, Löwenmark T, Rutegård J, Gunnarsson U, Strigård K (2017). Quality of life in patients with a permanent stoma after rectal cancer surgery. Qual Life Res.

[22] García-Rodríguez MT, Barreiro-Trillo A, Seijo-Bestilleiro R, González-Martin C. Sexual dysfunction in ostomized patients: a systematized review. Healthcare (Basel). 2021;9(5). 10.3390/healthcare9050520 10.3390/healthcare9050520 PMC8145568 33946735

[23] Noblit GW, Hare RD. Meta-Ethnography: Synthesizing Qualitative Studies. Beverly Hills: Sage; 1988.

[24] Sattar R, Lawton R, Panagioti M, Johnson J (2021). Meta-ethnography in healthcare research: a guide to using a meta-ethnographic approach for literature synthesis. BMC Health Serv Res.

[25] France EF, Cunningham M, Ring N (2019). Improving reporting of meta-ethnography: the eMERGe reporting guidance. BMC Med Res Methodol.

[26] Page MJ, McKenzie JE, Bossuyt PM (2021). The PRISMA 2020 statement: an updated guideline for reporting systematic reviews. Int J Surg.

[27] Kandemir D, Oskay U (2017). Sexual problems of patients with urostomy: a qualitative study. Sex Disabil.

[28] Sarabi N, Navipour H, Mohammadi E (2017). Sexual performance and reproductive health of patients with an ostomy: a qualitative content analysis. Sex Disabil.

[29] Campbell R, Pound P, Morgan M, et al. Evaluating meta ethnography: systematic analysis and synthesis of qualitative research. 2012. 10.3310/hta15430. 10.3310/hta15430 22176717

[30] Britten N, Campbell R, Pope C, Donovan J, Morgan M, Pill R (2002). Using meta ethnography to synthesise qualitative research: a worked example. J Health Serv Res Policy.

[31] Atkins S, Lewin S, Smith H, Engel M, Fretheim A, Volmink J (2008). Conducting a meta-ethnography of qualitative literature: lessons learnt. BMC Med Res Methodol.

[32] Manderson L (2005). Boundary breaches: the body, sex and sexuality after stoma surgery. Soc Sci Med.

[33] Paula MA, Takahashi RF, Paula PR (2012). Experiencing sexuality after intestinal stoma. J Coloproctol.

[34] Cardoso DBR, Almeida CE, de Santana ME, de Carvalho DS, Sonobe HM, Sawada NO (2015). Sexuality of people with intestinal ostomy. Rev Rene.

[35] Vural F, Harputlu D, Karayurt O (2016). The impact of an ostomy on the sexual lives of persons with stomas. J Wound Ostomy Continence Nurs.

[36] Kimura CA, Guilhem DB, Kamada I, De Abreu BS, Fortes RC (2017). Oncology ostomized patients’ perception regarding sexual relationship as an important dimension in quality of life. J Coloproctology.

[37] Dames NB, Squire SE, Devlin AB (2021). ‘Let's talk about sex’: a patient-led survey on sexual function after colorectal and pelvic floor surgery. Colorectal Dis.

[38] Capilla-Díaz C, Bonill-de Las Nieves C, Hernández-Zambrano SM (2019). Living With an Intestinal Stoma: A Qualitative Systematic Review. Qual Health Res.

[39] Yilmaz E, Çelebi D, Kaya Y, Baydur H (2017). A descriptive, cross-sectional study to assess quality of life and sexuality in Turkish patients with a colostomy. Ostomy Wound Manage.

[40] Albaugh JA, Tenfelde S, Hayden DM (2017). Sexual Dysfunction and Intimacy for Ostomates. Clin Colon Rectal Surg.

[41] Duluklu B, Çelik SŞ (2019). Effects of lavender essential oil for colorectal cancer patients with permanent colostomy on elimination of odor, quality of life, and ostomy adjustment: a randomized controlled trial. Eur J Oncol Nurs.

[42] DuHamel K, Schuler T, Nelson C (2016). The sexual health of female rectal and anal cancer survivors: results of a pilot randomized psycho-educational intervention trial. J Cancer Surviv.

[43] Du X, Wang D, Du H, Zou Q, Jin Y (2021). The correlation between intimate relationship, self-disclosure, and adaptability among colorectal cancer enterostomy patients. Medicine (Baltimore).

[44] Li J, Luo X, Cao Q, Lin Y, Xu Y, Li Q (2020). Communication needs of cancer patients and/or caregivers: a critical literature review. J Oncol.

[45] Chang YC, Chang SR, Chiu SC (2019). Sexual problems of patients with breast cancer after treatment: a systematic review. Cancer Nurs.

[46] Fuoto A, Turner KM (2019). Palliative care nursing communication: an evaluation of the COMFORT Model. J Hosp Palliat Nurs.

[47] Beach WA, Buller MK, Dozier DM, Buller DB, Gutzmer K (2014). The Conversations About Cancer (CAC) project: assessing feasibility and audience impacts from viewing The Cancer Play. Health Commun.

[48] Azar M, Kroll T, Bradbury-Jones C (2022). How do nurses and midwives perceive their role in sexual healthcare?. BMC Womens Health.

[49] Ertem G, CandanDonmez Y, Bilge A (2017). The journey of lıfe satisfaction from sexual life in breast cancer. Gumushane Univ J Health Sci.

[50] Reese JB, Beach MC, Smith KC (2017). Effective patient-provider communication about sexual concerns in breast cancer: a qualitative study. Support Care Cancer.

[51] Fatehi S, Maasoumi R, Atashsokhan G, Hamidzadeh A, Janbabaei G, Mirrezaie SM (2019). The effects of psychosexual counseling on sexual quality of life and function in Iranian breast cancer survivors: a randomized controlled trial. Breast Cancer Res Treat.

[52] Tat S, Doan T, Yoo GJ, Levine EG (2018). Qualitative exploration of sexual health among diverse breast cancer survivors. J Cancer Educ.

[53] Zhu X, Chen Y, Tang X (2017). Sexual experiences of chinese patients living with an ostomy. J Wound Ostomy Continence Nurs.

[54] Meira IFdA, Silva FRd, Sousa ARd, Carvalho ESdS, Rosa DdOS, Pereira Á. Repercussions of intestinal ostomy on male sexuality: an integrative review. Revista Brasileira de Enfermagem. 2020;73. 10.1590/0034-7167-2019-0245. 10.1590/0034-7167-2019-0245 32785513

[55] Thompson JC, Rogers RG (2016). Surgical management for pelvic organ prolapse and its impact on sexual function. Sex Med Rev.

[56] Park ER, Norris RL, Bober SL (2009). Sexual health communication during cancer care: barriers and recommendations. Cancer J.

[57] Sörensson M, Asplund D, Matthiessen P (2020). Self-reported sexual dysfunction in patients with rectal cancer. Colorectal Dis.

[58] Rash JK, Seaborne LA, Peterson M, Kushner DM, Sobecki JN (2023). Patient reported improvement in sexual health outcomes following care in a sexual health clinic for women with cancer. Support Care Cancer.

