# Supplementary material for: The sexuality experience of stoma patients: a meta-ethnography of qualitative research
Source: BMC Health Serv Res. 2023 May 16;23:489. doi: 10.1186/s12913-023-09532-2 (PMC10186813; doi:10.1186/s12913-023-09532-2)
Supplement: Supplementary file 1 — Additional file 1. [file 12913_2023_9532_MOESM1_ESM.docx]

**Supplementary File 1:**

**Search strategy for PubMed:**

(Ostomy [Mesh] OR “Surgical Stoma” [Mesh] OR Ostom* [Title/Abstract] OR Stoma* [Title/Abstract] OR “Stoma, Surgical” [Title/Abstract] OR “Surgical Stoma” [Title/Abstract] OR “Stomata, Surgical” [Title/Abstract] OR “Surgical stomata” [Title/Abstract] OR “Stomas, surgical” [Title/Abstract]) AND (“Sexual Behavior” [Mesh] OR “Sexual health” [Mesh] OR Sexuality [Mesh] OR sexualit*[Title/Abstract] OR “sexual behavior” [Title/Abstract] OR “Behavior, Sexual” [Title/Abstract] OR “Sexual Activity* ” [Title/Abstract] OR “Activity*, Sexual” [Title/Abstract] OR “Sex behavior” [Title/Abstract] OR “Behavior, sex” [Title/Abstract] OR “sexual health” [Title/Abstract] OR “health, sexual” [Title/Abstract] OR “sexual life” [Title/Abstract] OR “sexual experience” [Title/Abstract] OR “sexual function” [Title/Abstract]) AND (“Qualitative Research” [Mesh] OR “Evaluation Studies as Topic” [Mesh] OR “Interviews as Topic” [Mesh] OR “Focus Groups” [Mesh] OR “Grounded Theory” [Mesh] OR “qualitative research” [Title/Abstract] OR “evaluation studies as Topic” [Title/Abstract] OR “Interviews as Topic” [Title/Abstract] OR “focus groups” [Title/Abstract] OR “Grounded theory” [Title/Abstract] OR qualitative [Title/Abstract] OR interview* [Title/Abstract] OR “focus group* ”[Title/Abstract] OR “grounded theory” [Title/Abstract] OR phenomenolog* [Title/Abstract] OR narrative [Title/Abstract] OR “thematic analysis” [Title/Abstract])
